# Transcriptomic Analysis Reveals the Regulatory Mechanism of Color Diversity in *Rhododendron pulchrum* Sweet (Ericaceae)

**DOI:** 10.3390/plants12142656

**Published:** 2023-07-15

**Authors:** Nanyan Zhu, Chunhua Zhou

**Affiliations:** 1 College of Animal Science and Technology, Yangzhou University, 30 Wenhui East Rd., Yangzhou 225009, China; dx120200148@stu.yzu.edu.cn; 2 College of Horticulture and Landscape Architecture, Yangzhou University, 30 Wenhui East Rd., Yangzhou 225009, China

**Keywords:** *Rhododendron pulchrum*, transcriptome analysis, qRT-PCR, anthocyanin, gene expression

## Abstract

*Rhododendron pulchrum* Sweet is a traditional ornamental plant cultivated in China and presents a great variation in petal coloration. However, few studies have been performed to reveal the genes involved and the regulatory mechanism of flower color formation in this plant. In this study, to explore the underlying genetic basis of flower color formation, transcriptome analysis was performed by high-throughput sequencing techniques on four petal samples of different colors: purple, pink, light pink, and white. Results show that a total of 35.55 to 40.56 million high-quality clean reads were obtained, of which 28.56 to 32.65 million reads were mapped to the reference genome. For their annotation, 28,273, 18,054, 24,301, 19,099, and 11,507 genes were allocated to Nr, Swiss-Prot, Pfam, GO, and KEGG databases, correspondingly. There were differentially expressed genes among the four different petal samples, including signal-transduction-related genes, anthocyanin biosynthesis genes, and transcription factors. We found that the higher expressed levels of genes associated with flavonol synthase (FLS) might be the key to white formation, and the formation of red color may be related to the higher expression of flavanone 4-reductase (DFR) families. Overall, our study provides some valuable information for exploring and understanding the flower color intensity variation in *R. pulchrum*.

## 1. Introduction

*Rhododendron* is the largest genus belonging to the family Ericaceae, which is known for colorful flowers [1]. In recent years, varieties of *Rhododendron* have been grown as ornamental plants, making them become the most popular evergreen shrubs all over the world [2]. As one of the important characteristics of *Rhododendron*, color has long been a concern of breeders and consumers. The author (N.Z.) and her team found that there were a few white and pink flowers in the purple *Rhododendron pulchrum* Sweet community. Petals are an extremely essential part of numerous ornamental plants, and differences in petal color, tones, and intensity directly influence the ornamental value of a plant [3]. Previous studies have shown that flower color is attributed to specific pigments in petal cells, including flavonoids, carotenoids, and alkaloids [4,5]. In addition, flavonoids are major pigments in flowers that are responsible for the coloration of plant petals [6,7]. Flavonoids are naturally occurring polyphenols in plants. According to their structural characteristics, flavonoids are usually classified into anthocyanins and flavonoid alcohols [8,9]. Among them, anthocyanins are the main water-soluble pigments in flowers that constitute the color of plant petals. They are mainly accumulated in the vacuoles of petal epidermal cells and impart to the petals a colorful appearance from light pink to purple [10]. Color modification of flowers can be achieved by enhancing the accumulation of anthocyanins [11]. Flavonoids, on the other hand, have auxiliary effects on anthocyanins. The petals of plants with a higher concentration of flavonoids usually show bright colors [12]. In general, anthocyanins are synthesized through the secondary metabolic pathway, which occurs in a wide range of plants, such as *Gerbera hybrida* and *Triticum aestivum* [13,14]. However, the molecular mechanisms regulating anthocyanin synthesis have not been elucidated in *R. pulchrum* due to the structural diversity of anthocyanins among the different plant species [15].

Anthocyanins are derived from the branches of the flavonoid biosynthesis pathway, which also leads to the production of isoflavonoids and flavonols. The anthocyanin biosynthesis pathway has been extensively studied in a range of plant species and involves several enzymes encoded by different structural genes [16,17]. First, phenylalanine ammonia-Lyase (PAL) catalyzes the deamination of phenylalanine to cinnamic acid in the initial step of the flavonoid pathway [18], while chalcone synthase (CHS) catalyzes the synthesis of naringenin chalcone with 4-coumaroyl CoA and malonyl CoA as substrates in the first committed step of flavonoid biosynthesis [19]. Subsequently, chalcone isomerase (CHI) catalyzes the conversion of naringenin chalcone to naringenin [20,21]. The naringenin is then catalyzed to dihydrokaempferol (DHK) via flavanone-3-hydroxylase (F3H). Moreover, DHK can be further hydroxylated by flavonoid 3′-hydroxylase (F3′H) to produce dihydroquercetin (DHQ). In the third stage, all obtained DHQ are reduced to leucocyanidin by dihydroflavonol 4-reductase (DFR) and then are further converted into anthocyanidins by anthocyanidin synthase (ANS). Of the main key enzymes discovered, CHS catalyzes the first step of flavonoid biosynthesis, and DFR is the first committed enzyme of anthocyanin biosynthesis [22].

It is known that the MBW complexes (MYB-*bHLH*-WD40) consist of the MYB transcription factors (TFs), *bHLH* TFs, and one WD-40 repeat factor (WDR) [23], which play important roles in regulation of the expression of the genes involved in the anthocyanin biosynthetic pathway at the transcriptional level [24]. The MYB regulator is a large family of proteins with diverse functions, and most of the MYB genes in plants belong to *R2R3-MYB* TFs. Several studies have shown that *R2R3-MYB* TFs control the transcriptional regulation of anthocyanin structural genes [25]. Another crucial TF regulating anthocyanin biosynthesis is the *bHLH* protein, which is critical for the activity of *R2R3-MYB*. For example, the *bHLH* TF in *Arabidopsis* interacts with the *R2R3-MYB* protein to regulate DFR gene expression in anthocyanin biosynthesis [26]. It was considered that the WDR proteins could interact with different *R2R3-MYB* and *bHLH* to form transcription complexes, which play vital roles in anthocyanin accumulation in vegetative tissues [27].

Currently, to our best knowledge, research on flower coloration in *R. pulchrum* is very limited, and the molecular mechanism regulating the flower coloration remains unknown. It would help to clarify the mechanisms controlling anthocyanin accumulation through the analysis of the expression pattern of the key genes related to color formation in *Rhododendron*. In recent years, with the development of next-generation sequencing, transcriptome sequencing (RNA-seq) has been widely used to identify differentially expressed genes (DEGs) in many plants [28,29]. Therefore, transcriptome sequencing of *R. pulchrum* flowers will provide meaningful knowledge to unravel the molecular mechanism of color formation. In this current study, four varieties with different petal colors were used as the experimental materials to determine the genotypic difference at the transcriptional level using RNA-seq technology. Our findings will provide a useful resource for further analyzing the molecular mechanism of color formation and intensity of *R. pulchrum*.

## 2. Results

### 2.1. The Contents of Anthocyanins Components and Total Flavonoids

**Figure 1:**
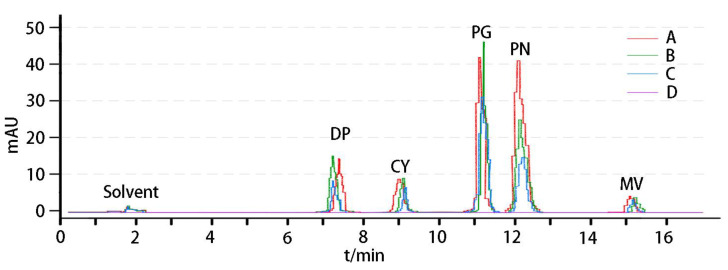
High-performance liquid chromatograms (HPLC) of mixed anthocyanins in petals. The *y*-axis represents the peak area. DP, delphinidin; CY, cyanidin; PG, pelargonidin; PN, peonidin; MV, malvidin.

The petals of *R. pulchrum* have deep-color blotches in the center. The components and contents of anthocyanins both in petals and blotches were analyzed by HPLC and compared with the standard. The results show that the peak value of pigments appeared in samples A, B, and C around 7.32, 9.07, 10.55, 12.30, and 15.6 min, which is consistent with the detection time of five pigment standards (delphinidin, 6.95; cyanidin, 8.91; pelargonidin, 10.82; peonidin, 11.95; malvidin, 14.80 min) (Figure 1). In petal samples, peonidin was the main anthocyanin in sample A. The content of peonidin in B and C decreased significantly, and pelargonidin pigment became the main anthocyanin that determined the color of samples B and C (Table S1). The main anthocyanins in the deep-color blotches were peonidin. Anthocyanin components were not detected in the petals of D, and only a small amount of anthocyanins were detected in the blotches. The analysis of flavonoid content in sample petals significantly showed that the lighter the color of the petals, the higher the flavonoid content, and there was no significant difference in the flavonoid content in the dark spots of the petals (Table 1).

### 2.2. Overview of RNA-Seq Data and Sequence Assembly

To further research the molecular mechanism of *R*. *pulchrum* petal coloration, transcriptome analysis was performed via RNA-seq. Libraries were prepared from three biological replicates of each variety, and twelve libraries were established. The cDNA libraries were then submitted for transcriptome sequencing analysis on the Illumina HiSeq 2000 platform. A total of 38.67 to 43.72 million raw sequencing reads were generated for each variety (Table 2). After removing low-quality reads, 35.55 to 40.56 million high-quality clean reads were obtained, which accounted for over 90% of the raw reads. The percentage of bases with Q30 (high sequencing quality) was not less than 89.80%. The clean reads were then aligned to the corresponding reference genome using Hisat2 (version 2.1.0) software. The number of mapped reads ranged from 28.56 to 32.65 million, and the mapping ratio of each sample ranged between 79.32 and 80.66%. In addition, the percentage of reads that were only aligned to one position (uniquely mapped reads) was greater than 94.70% (Table 2).

**Figure 2:**
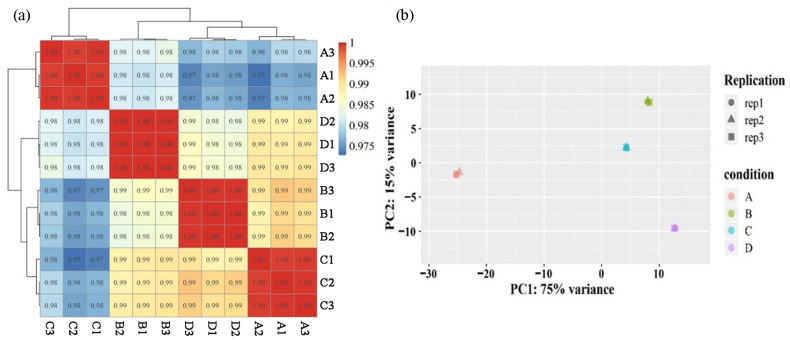
Different samples analysis. (**a**) Pearson correlation coefficient of biological replicates of different samples. The correlation coefficient between two samples was calculated based on the FPKM values of those samples. The left and upper sides of the figure show sample clustering, and the right and lower sides show sample names. (**b**) Principal component analysis (PCA) of the similarities and differences between the four samples used for RNA-seq.

The abundance of each gene was calculated based on the fragments per kb per million reads (FPKM) method. Moreover, Pearson’s correlation test was applied to calculate the relation of expression patterns among the different samples. The gene expression levels showed similar patterns within sample groups and differences between groups (Figure 2a), indicating that the analysis results are reliable. Principal component analysis (PCA) was used to further explore the differences and similarities, which showed a clear separation among the four varieties with different colors. Meanwhile, the biological replicates of each sample were clustered together, indicating a high degree of transcriptional similarity (Figure 2b). Collectively, these results show that the RNA sequencing quality was suitable for further analysis.

### 2.3. Annotation of R. pulchrum Transcriptome

Five public databases were used for annotation of the unigenes in *R*. *pulchrum*. The results show that a total of 32,999 unigenes were annotated according to the BLASTx results, of which 28,273, 18,054, 24,301, 19,099, and 11,507 genes could be annotated using the Nr database (85.68%), Swiss-Prot database (54.71%), Pfam database (73.64%), GO database (57.88%), and KEGG database (34.87%), respectively (Table 3).

### 2.4. Differentially Expressed Genes Analysis

**Figure 3:**
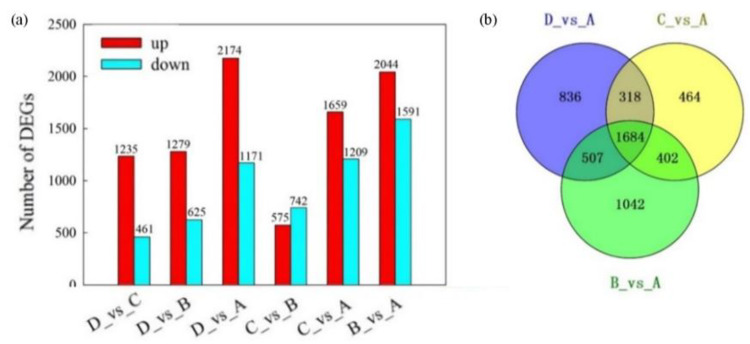
Number of differentially expressed genes (DEGs) in pairwise comparisons of the four varieties. (**a**) The total number of DEGs is up-regulated and down-regulated. The red and cyan colors indicate the up-regulated and down-regulated genes, respectively. (**b**) Venn diagram of DEGs for making a comparison among the three groups.

In the current study, differentially expressed genes (DEGs) were selected using a cut-off value of |log_2_FoldChange| ≥ 1 and an adjusted *p*-value < 0.05. Thousands of DEGs were identified through pairwise comparisons (Figure 3a). Among them, the B vs. A group had the largest number of DEGs, followed by the D vs. A and C vs. A groups. It is worth noting that the number of up-regulated genes was significantly higher than that of down-regulated genes, with only one exception: the C vs. B comparison. Interestingly, the largest difference between the numbers of DEGs was observed in the D vs. A comparison, reaching a total of 1003 genes. Furthermore, the common DEGs among the D vs. A, C vs. A, and B vs. A groups were selected using the Venn Diagram Plotter tool (http://omics.pnl.gov/software/venn-diagram-plotter (accessed on 22 January 2022)). The results indicate that there were 1684 genes found to be differentially expressed in the three-way comparisons (Figure 3b), which suggests that these DEGs might have different functions, resulting in different petal color pattern forms.

**Figure 4:**
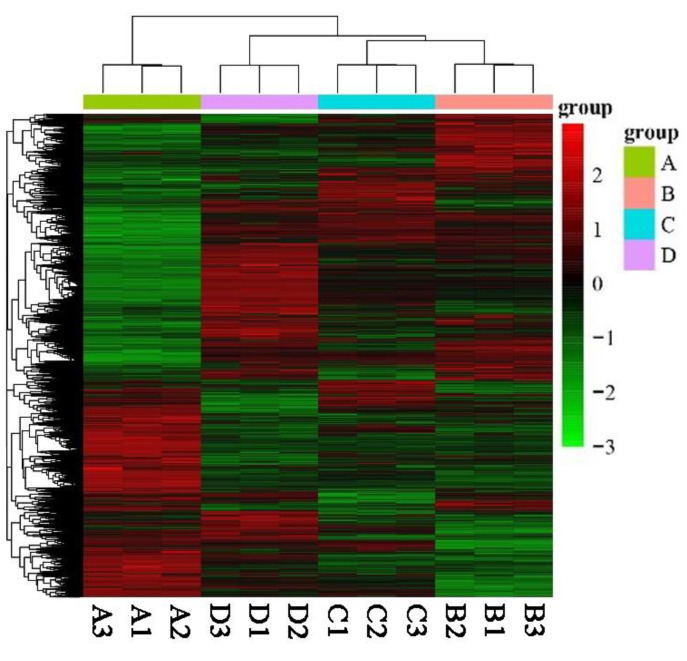
Heatmap showing the relative expression profiles of DEGs by Euclidean distance among the four samples. A selection of genes is shown in rows, and each column represents an individual sample. The upper dendrogram showed gene clustering by complete linkage, while the left showed clustering of gene expression quantity. The filled colors indicate the gene expression levels (from low—green to high—red).

To explore the differences in gene expression patterns, we performed a hierarchical clustering analysis in a heatmap. Overall, three main clusters of samples were identified (Figure 4). The first cluster mainly consisted of A samples, and cluster two mainly consisted of D samples, whereas cluster three comprised B and C samples. The smallest number of up- and down- regulated DEGs was identified in the C vs. B comparison (Figure 3a), which implied transcriptional similarity between B and C samples. However, the contents of anthocyanins between B and C were distinctly different (Table S1). Therefore, we may assume that the 167 unique up-regulated genes in B samples might be related to the anthocyanin biosynthetic pathway. Meanwhile, we found that the expression patterns of most DEGs were opposite between A and D samples.

**Figure 5:**
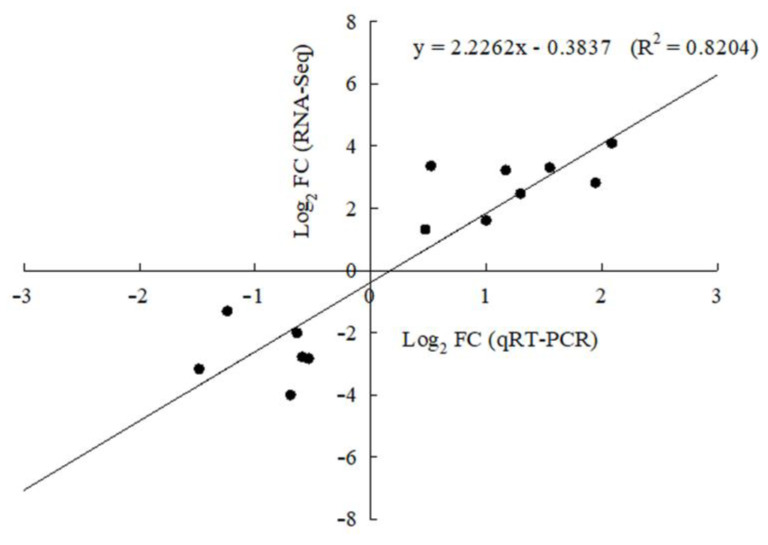
Regressions of gene expression ratio between RNA-seq and qRT-PCR with GAPDH as a reference. The *x*-axis represents relative gene expression fold change obtained by qRT-PCR, and the *y*-axis represents relative gene expression fold change obtained by RNA-seq. Each point represents the average of the three biological replicates.

To verify the reliability of the RNA-seq analysis, we randomly selected several genes for validation using quantitative RT-PCR (qRT-PCR). All the primers used in this study are provided in Table S2, and these genes are responsible for L-ascorbate oxidase, pectin esterase, and beta-glucuronosyltransferase. The results show that the coefficient of determination (R^2^) between the qRT-PCR data and RNA-seq data was 0.82 (Figure 5), which indicates the high reliability of the RNA-seq results in our study.

### 2.5. Classification of GO and KEGG Terms

With gene ontology (GO) annotation, DEGs were classified into three major functional categories: biological process (BP), cellular component (CC), and molecular function (MF). The three types of comparisons presented similar distribution patterns, while the numbers and types of enriched pathways were different (Figures S1 and S2). For the category of BP, the pathway “localization” (GO: 0051179)–“oligosaccharide transport” (GO: 0015772)–“sucrose transport” (GO: 0015770) had a higher number of genes than others (Figure S2a–c). Among the CC functions, the pathway “cell periphery” (GO: 0071944)–“plasma membrane” (GO: 0005886)–“obsolete intrinsic component of plasma membrane” (GO: 0031226) presents the higher number of DEGs (Figure S2d–f). In the MF category, most of the DEGs were mapped to “nutrient reservoir activity” (GO: 0045735) and “catalytic activity” (GO: 003824) (Figure S2g–i).

GO terms with a corrected *p*-value < 0.05 were considered to be significantly enriched. For simplicity, only the top twenty most significant GO terms were selectively presented in each pairwise comparison. GO analysis revealed that most enriched GO terms were assigned to molecular function, followed by biological process (Figure S3 and Table S3). In the D vs. A comparison, these DEGs were associated with six BP terms, five CC terms, and nine MF terms. Comparison of the C sample with the A sample revealed that there were six, five, and nine functional terms assigned to the main categories of three ontologies. Among the 20 GO terms, 2, 3 and 15 GO terms belonged to these 3 functional terms in the B vs. A comparison. Notably, we found that most of the significantly enriched GO MF terms contain genes involved in “sucrose transmembrane transporter activity” (GO: 008515) and “disaccharide transmembrane transporter activity” (GO: 0015154).

**Figure 6:**
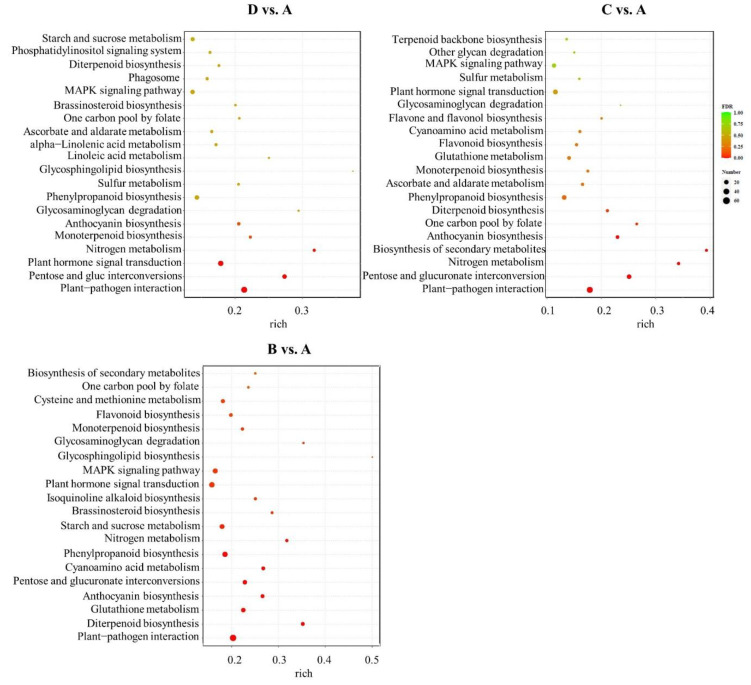
KEGG pathway enrichment analysis of the identified DEGs among the three comparison groups. The *x*-axis represents the rich factor, the *y*-axis indicates the name of the KEGG pathway. The size of the dot indicates the number of DEGs in each pathway, while dot color represents the *q*-value of the enrichment.

The KEGG enrichment analysis was performed to further explore the various metabolic and biosynthesis pathways of the DEGs obtained in different comparisons. The top 20 most significantly enriched pathways that were associated with the DEGs are listed in Figure 6. For example, “Plant hormone signal transduction”, “Phenylpropanoid biosynthesis”, and “Anthocyanin biosynthesis” were significantly enriched in the D vs. A comparison. “Biosynthesis of secondary metabolites” and “Anthocyanin biosynthesis” were most highly enriched in the C vs. A comparison. “Glutathione metabolism”, “Anthocyanin biosynthesis”, and “Phenylpropanoid biosynthesis” were detected in the B vs. A comparison. In particular, pathways related to “phenylpropanoid biosynthesis” (ko00940), and “Anthocyanin biosynthesis” (ko00942) were all significantly enriched in the three pairwise comparisons.

### 2.6. The Key DEGs Involved in Anthocyanin Biosynthesis Pathway

Since the contents of anthocyanin components in four peals were correlated with color intensity (Table S1), to investigate the differences in anthocyanin biosynthesis in the petals of the four varieties, DEGs in the anthocyanin biosynthesis pathway were detected, including the phenylpropanoid biosynthesis, flavonoid biosynthesis, and anthocyanin biosynthesis pathways (Table 4). Phenylalanine is the major amino acid precursor for phenylpropanoids. In our study, the gene (*Rhsim04G0145800*) encoding phenylalanine ammonia-Lyase (PAL) was down-regulated in the A sample relative to the B and D samples. Similarly, one gene (*Rhsim01G0211600*) linked to 4-coumarateCoA ligase (4CL) in converting 4-coumaric acid to 4-coumaroyl-CoA was down-regulated in the A sample, as compared to the C and D samples. Additionally, several enzymes were identified, such as flavonoid 3′,5′-hydroxylase (F3′5′H), flavonol synthase (FLS), flavanone 4-reductase (DFR), and anthocyanidin synthase (ANS), which were associated with two, six, three, and one DEGs, respectively. It has been noticed that six genes (*RhsimUnG0095500*, *RhsimUnG0143300*, *RhsimUnG0134400*, *Rhsim04G0219900*, *RhsimUnG0007900*, *RhsimUnG0105700*) encoding FLS were highly expressed in the B, C, and D samples, especially in the D sample. These results suggest that the genes encoding FLS might be the key to absence of pigments in white petals, leading to more dihydroflavonol entering the flavonols branch. In contrast, three genes in the A sample associated with DFR (*Rhsim06G0030600*, *Rhsim06G0030500*, and *Rhsim06G0030400*) were more highly expressed than in the B, C, or D samples (Table 4). These results indicate that the genes encoding DFR might be critical for color formation, due to causing more dihydroflavonols to enter into the anthocyanin pathway. Interestingly, DFR in the B sample compared to the A sample showed a greater degree of up-regulation than in the C sample, and the high expression of ANS (*Rhsim07G0096600)* in the B sample may explain the similarity of anthocyanins content between A and B samples.

As we know, anthocyanins and flavonoids are mainly pigments in flowers. In this current study, DEGs involved in the anthocyanin biosynthesis pathway were detected according to KEGG pathway analysis. In total, 17 genes were detected in the D vs. A comparison. Compared with the D sample, 12 genes were up-regulated and 5 genes were down-regulated in the A sample. Correspondingly, 19 DEGs were detected between C and A samples, including 14 up-regulated genes and 5 down-regulated genes in the A sample. Additionally, 22 genes were identified in the B vs. A comparison, of which 15 genes were up-regulated and 7 genes down-regulated in the A sample (Table S4). The overlaps among the three pairwise comparisons were calculated, and 11 DEGs were found in the overlapping regions. In addition, eight genes were up-regulated in the A sample relative to the B, C, and D samples, including one, two, and five DEGs encoding UGAT, 5AT, and UGT75C1, respectively (Table S4).

### 2.7. Identification of Transcription Factors Regulating Petal Color Formation

**Figure 7:**
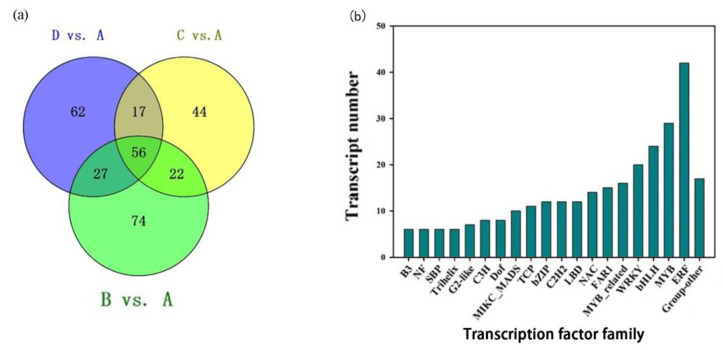
Differentially expressed TFs in the three pairwise comparisons. (**a**) Distribution of the TF families that include more than 10 DEGs. (**b**) Venn diagram of differentially expressed TFs in D vs. A, C vs. A, B vs. A comparisons.

Transcription factors (TFs) are important regulators that play key roles in regulating the expression of the genes during anthocyanin biosynthesis in flowering plants [30]. According to the annotation in Plant TF Database v4.0, a total of 302 TFs from 36 families were identified in the *R. pulchrum*, of which 162, 139, and 179 TFs were found in the comparisons of D vs. A, C vs. A, and B vs. A (Figure 7a and Table S5). Most of the genes encoding TFs were down-regulated in the A sample relative to the B, C, and D samples. In the D vs. A comparison, 101 TFs were up-regulated and 61TFs were down-regulated. In the C vs. A comparison, 95 TFs were up-regulated and 44 TFs were down-regulated. In the B vs. A comparison, 96 TFs were up-regulated and 83 TFs were down-regulated. Moreover, 11 TF families containing more than 10 DEGs were identified: ERF (42), MYB (29), *bHLH* (24), WRKY (20), MYB related (16), FAR1 (15), NAC (14), LBD (12), C2H2 (12), bZIP (12), and TCP (11) (Figure 7b). Notably, the ERF, MYB, *bHLH*, and WRKY families were relatively large TF families in the three pairwise groups (Table S5).

Furthermore, we investigated ERF, MYB, and *bHLH* TFs to enhance our understanding of their involvement in regulating color formation in *R*. *pulchrum*. The overlaps among the three pairwise comparisons were calculated, and 56 TFs were found in the overlapping regions (Table S5). In our present study, five DEGs (*Rhsim02G0040100*, *Rhsim03G0176600*, *Rhsim07G0072500*, *Rhsim08G0214100*, *Rhsim12G0192100*) encoding ERF and three DEGs (*Rhsim03G0180500*, *Rhsim06G0163100*, *Rhsim09G0042000*) encoding MYB were up-regulated in the D vs. A, C vs. A, and B vs. A comparisons. Moreover, the expression level of one gene (*Rhsim08G0230000*) linked to *bHLH* was up-regulated in the A sample, as compared to the B, C, and D samples (Table S5).

### 2.8. DEGs Related to Hormone Signaling

Plant hormones (IAA, abscisic acid, ethylene, brassinosteroids, etc.) play a direct role in the biosynthesis of anthocyanins [31,32]. KEGG analysis showed that there were differences in phytohormone signaling transduction in the three pairwise comparisons. To thoroughly analyze the molecular mechanism of anthocyanin transformation, we investigated the gene expression differences in hormone signaling in the three pairwise groups. The results show that most of the genes involved in phytohormone metabolic pathways were down-regulated in the A sample relative to the B, C, and D samples. In the D vs. A comparison, 42 hormone-signaling-related DEGs were up-regulated and 17 DEGs were down-regulated. Meanwhile, 38 DEGs were screened in the C vs. A comparison, with 22 up-regulated and 16 down-regulated. For the comparison of B vs. A, 52 DEGs revealed differences, including 30 up-regulated and 22 down-regulated genes. (Table S6).

The overlapping DEGs among the three pairwise comparisons were screened using a Venn diagram (Figure S4). The results indicate that 21 genes were differentially expressed, and we found that the genes involved in auxin (IAA) or brassinosteroid (BR) signaling were the most enriched (Table S6). Three DEGs (*RhsimUnG0255100*, *Rhsim06G0190800*, *Rhsim13G0033000*) involved in IAA signaling were identified, and the abundance of these transcripts was significantly decreased in the A sample relative to the B, C, and D samples (Table S6). Moreover, one (*Rhsim07G022810*) and eight DEGs (*Rhsim09G0181000*, *Rhsim07G0028200*, *RhsimUnG0200100*, etc.) involved in ABA and BR signaling, respectively, were found in the three comparison groups. Interestingly, these genes were more lowly expressed in the A sample relative to the B, C, and D samples.

## 3. Discussion

Anthocyanins and flavonoids are the key pigments that determine flower colors. In general, cyanidin, pelargonidin, and paeoniflorin are beneficial to the formation of red color; delphinium and malvidin are conducive to the formation of blue color [9,33]. The combined effect of these pigments makes the flowers of *Rhododendron* present white, pink, red, purple, and other colorful colors. The results of HPLC show that the main anthocyanins in different samples were the same, but the content of each pigment was significantly different. It is inferred that the color depth of *R. pulchrum* is related to the content of main anthocyanins in the petals. The high content of peonidin in sample A may be an important factor in the formation of deep color. Peng et al. also put forward a similar conclusion that the anthocyanin biosynthesis pathway is the main metabolic pathway of flower color formation in their study on *Hydrangea macrophylla* cv. ‘Forever Summer’ [34]. The biosynthesis and floral color regulation mechanism of flavonoids in *R. pulchrum* was elaborated in Xia et al.’s research [12]. In the four samples of this experiment, flavonoid content was negatively correlated with colors. This shows that the color depth of *R. pulchrum* is produced by the combined action of anthocyanins and flavonoids, which is consistent with the research results of Liu et al. [35].

The intensity of petal color change was correlated with the concentration of anthocyanin in plants [36]. Xue [37] reported that the anthocyanin content increased as the flower color deepened, and the expression of key structural genes was significantly increased in red-flowered strawberry in comparison with white-flowered strawberry. Previous results suggested that the competition expression of FLS and DFR genes led to the redirection of flavonol and anthocyanin accumulation [38,39]. In this study, the difference in gene expression was consistent with the accumulation of anthocyanins in different samples. This discovery is like that of blueberry fruit development, in which a partial correlation between anthocyanin-related gene expression and anthocyanin concentration was found [40]. Meanwhile, transcriptome analysis revealed that the FLS and DFR genes were differentially expressed in the flower color of the four different varieties. Six genes encoding FLS were highly expressed, especially in the D sample, and three genes in the A sample associated with DFR were more highly expressed than in the B, C, or D samples. These results indicate that the expression levels of genes encoding DFR were higher in deeper color flowers, which significantly promoted the conversion of dihydroflavonols to anthocyanins. On the contrary, the increased expression of FLS in the white flowers promoted the conversion of dihydroflavonols into flavonols (Table 4). There may be substrate competition between FLS and DFR, controlling the metabolic flux via the branching of the flavonoid biosynthetic pathway, resulting in changes in flower color intensity in *R. pulchrum*.

Transcription factors perform important roles in all plants. In the current study, most of the TFs were enriched in ERF, MYB, *bHLH*, WRKY, MYB related, FAR1, and NAC families during anthocyanin transformation (Figure 7b). ERF plays a particularly important role in the color expression of light petals, especially pink petals, by affecting the upstream pathway of anthocyanin biosynthesis [41]. The class of TFs was previously involved in the regulation of petal color formation [42]. The *bHLH* proteins, forming one of the largest TF families, play vital roles in various metabolic, physiological, and developmental processes in plants [43]. In our study, one gene (*Rhsim08G0230000*) encoding *bHLH* was more highly expressed in the A sample, indicating that this *bHLH* was a positive regulator of anthocyanins biosynthesis in *R. pulchrum.* It has been reported that MYB is closely related to flavonoid metabolic pathways [44]. However, some studies showed that there were also inhibitory factors in the MYB family that inhibit anthocyanin biosynthesis [45]. In our study, one gene (*Rhsim09G0042000*) encoding the MYB family members was significantly down-regulated in the D vs. A, C vs. A, and B vs. A comparisons (Table S5), which suggested it inhibited the anthocyanin accumulation.

Phytohormones are also critical internal factors affecting anthocyanin biosynthesis in many plant species. Wang et al. [46] reported that auxin inhibits anthocyanin accumulation and reduces the expression of genes linked to anthocyanin biosynthesis in apples. Based on our transcriptome data (Table S6), six genes associated with auxin-mediated signaling pathways were detected in the three groups, four of which were down-regulated in the A sample. In addition, the transcript level of two genes (*Rhsim09G0181000* and *Rhsim07G0028200*) encoding brassinosteroid was increased in the A sample. Similarly, Peng et al. [47] found that BR affects anthocyanin accumulation by regulating the anthocyanin biosynthesis genes in *Arabidopsis* seedlings. In addition, the response proteins in signal transduction of IAA and ABA were prominently regulated during anthocyanin transformation, and the functions of these hormone-responsive proteins deserve further investigation.

The results for anthocyanin composition in petals and deep-color blotches obtained by HPLC show that the accumulation of delphinidin and peonidin may be the pigment basis for the formation of deep-color blotches (Table S1). In the four samples, the down-regulation of DFR genes related to anthocyanin synthesis (Table 4) was inconsistent with the decreasing trend in anthocyanin contents. This is possibly due to the defect that petals and deep-color blotches were not sequenced separately for RNA-seq. The presence of deep-color blotches resulted in high expression of DFR genes in B and C samples. Studies of tree peony (*Paeonia suffruticosa*) have already shown that petal coloration and spot coloration are differentially regulated by MYB transcription factor [25,48]. Separating petals and deep-color blotches for independent analysis, revealing the transcriptional and metabolic regulatory mechanisms of petal and deep-color blotches formation, will be the focus of our subsequent research.

## 4. Materials and Methods

### 4.1. Plant Materials

**Figure 8:**
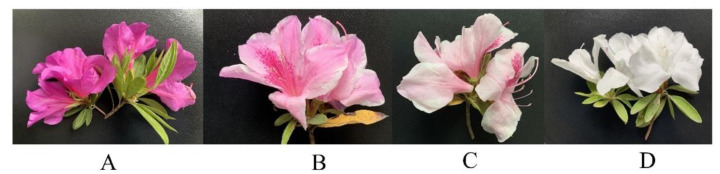
Color comparison among four phenotypes of *Rhododendron pulchrum* Sweet. The samples of purple, pink, light pink and white petals are labeled as (**A**–**D**).

*Rhododendron pulchrum* Sweet used in this study was cultivated at Yangzhou University (32°23′ N, 119°25′ E), which located in Jiangsu Province, China. The purple (purple petals, purple blotches) flower variety of *R. pulchrum* is “Zihe”, the pink (pink petals, purple blotches) and light pink (light pink petals, purple blotches) flower varieties are “Fenhe”, and the white (white petals, no or very few blotches) flower variety is “Baihe”. To explore the petal color regulation mechanism of *R. pulchrum*, we selected well-grown plants that had been planted for more than three years and collected full-bloom flowers having four different petal colors. The samples of purple, pink, light pink and white petals are labeled as A, B, C, and D, respectively (Figure 8). Fresh petals were collected on 24 April 2021, and flowers of the same color gradient from three individual plants were pooled as one sample. They were then frozen and ground in liquid nitrogen and subsequently stored in ultralow-temperature freezer under −80 °C for subsequent analysis.

### 4.2. Determination of Contents of Anthocyanins Components and Total Flavonoids

The frozen petal tissue (0.5 g) was added to 1.5 mL of 2 M HCl in methanol solution for grinding and homogenization, and low-temperature ultrasound for 15 min was followed by centrifugation [49]. The supernatant was used for anthocyanin extract. The contents and components of anthocyanins in the four samples were determined by high-performance liquid chromatography (HPLC), and chromatograms were mapped by chromatographic workstation software (LC-WS100) [50]. The total flavonoid content in each sample was measured by UV spectrophotometry [51]. The results are expressed as mg quercetin equivalent per g of dry weight (QE mg/g DW).

### 4.3. RNA Extraction and Transcriptome Sequencing

For the RNA-seq study, the petals of each variety were completely cut and mixed (including the deep-color blotches in the centers of the petals), and three biological replicates were conducted. Total RNA extraction was completed using the RNA simple Total RNA Kit (Tiangen, Shanghai, China) from petals following the manufacturer’s instructions. Extracted RNA purity was determined in a Nanodrop spectrophotometer (Thermo Scientific, Waltham, MA, USA), and RNA integrity was assessed using the Agilent 2100 Bioanalyzer (Agilent Technologies, Santa Clara, CA, USA). RNA-seq libraries were generated using the TruSeq RNA Sample Preparation Kit (Illumina, San Diego, CA, USA). After preparation, twelve libraries were sequenced on the Illumina HiSeq platform [52]. The RNA-seq analysis was performed by Panomix Biomedical Tech Co., Ltd. (Suzhou, China).

### 4.4. RNA-Seq Data Analysis

After removing adapter sequences, ploy-N-containing reads, and low-quality sequences from the raw data, the remaining clean reads were mapped to the reference genome via Hisat2 (version 2.1.0) software [53]. The expression levels of each gene were calculated and normalized as fragments per kilobase million (FPKM) measurements. Functional annotations of the identified transcripts were then aligned against various databases, including NR [54], Swiss-Prot [55], protein family (Pfam) [56], Gene Ontology (GO) [57], and Kyoto Encyclopedia of Genes and Genomes (KEGG) [58]. Differential gene expression analysis across varieties was performed using the DESeq2 package (version 1.16.1) [59], and genes with an adjusted *p*-value < 0.05 and absolute log_2_ fold changes (FC)  ≥  1 were set as the screening criteria for significantly differential expression. Based on the identified DEGs, three types of GO categories were obtained, including biological processes (BP), cellular components (CC), and molecular function (MF). Additionally, the functional enrichment analysis of GO and KEGG was implemented to identify which genes were significantly enriched. GO terms and KEGG pathway with a threshold of false discovery rate (FDR) < 0.05 were considered as the significance level.

### 4.5. Verification of RNA-Seq by qRT-PCR

To confirm the reliability of RNA-seq data, we randomly selected several genes and tested their expression profiles using qRT-PCR. Total RNA was isolated from petals as already described above. The first strand of complementary DNA (cDNA) was synthesized from total RNA using the cDNA synthesis kit (Vazyme Biotech, Nanjing, China). Finally, qRT-PCR was performed on the CFX96 real-time PCR system (Bio-Rad, Hercules, CA, USA) using the ChamQ Universal SYBR qPCR Master Mix (Vazyme Biotech, Nanjing, China) according to the manufacture recommended protocol. The amplification reaction volume was 20 µL, containing 10 µL of 2 × SYBR qPCR Master Mix and 10 µM of forward and reverse primers. All reactions were run in triplicate assays using the following cycling conditions: initial denaturation at 95 °C for 30 s followed by 40 cycles of PCR consisting of denaturation at 95 °C for 10 s and annealing at 60 °C for 30 s. All gene-specific primers for qRT-PCR were designed using the online primer design software (https://www.ncbi.nlm.nih.gov/tools/primer-blast/ (accessed on 22 January 2022)), and all the primers used in this study are presented in Table S2. The relative gene expression levels were calculated using the 2^−∆∆CT^ method [60].

### 4.6. Statistical Analyses and Bioinformatics

Data on the contents of anthocyanins and flavonoids were subjected to analysis of variance (one-way ANOVA) using SPSS 20.0 software (IBM Corp, Armonk, NY, USA), while graphs were produced using Microsoft Excel (v2013, Microsoft Corp., USA) and Origin 8.0 (Origin Lab Corporation, Northampton, MA, USA) [61]. Treatment means were presented as the mean ± standard deviations of the mean using the three replicates [62]. Bioinformatics data were analyzed using R statistical software package in R Studio version 0.99.446 (RStudio, Inc. 2015) [63].

## 5. Conclusions

In summary, we used transcriptome approaches to study the molecular mechanism of petal coloration of four varieties of *R. pulchrum*. Our results show that variation in the flower color was related to the expression level of anthocyanin biosynthesis genes. The higher expression levels of genes associated with FLS might be the key to white formation, leading to more dihydroflavonol entering the flavonols branch. The formation of red color may be related to the higher expression of DFR genes, which promoted the accumulation of anthocyanin. The structural genes and their regulators (TFs) in this research provided valuable molecular information on the flower color intensity variation in *R. pulchrum*.

## Figures and Tables

**Table 1:** The content of total flavonoids.

Sample	Total Flavonoids (QE mg/g DW)	
	Petal	Spot
A	1.14 ± 0.12 ^d^	1.03 ± 0.07 ^b^
B	1.71 ± 0.06 ^c^	1.03 ± 0.03 ^b^
C	1.97 ± 0.06 ^b^	1.44 ± 0.10 ^a^
D	2.24 ± 0.06 ^a^	1.57 ± 0.08 ^a^

Means are followed by ± standard deviations. Different lowercase letters indicate significant difference between treatments based on one-way ANOVA (*p* < 0.05). QE mg/g DW, mg quercetin equivalent per g of dry weight.

**Table 2:** Summary of the transcriptome sequencing dataset.

Sample	Raw Reads	Clean Reads	Q30 (%)	Total Mapped	Unique Mapped
A1	42,949,574	38,765,084 (90.25%)	91.04	31,266,555 (80.66%)	29,622,723 (94.74%)
A2	39,308,136	36,123,834 (91.89%)	90.85	29,098,513 (80.55%)	27,596,151 (94.84%)
A3	38,671,858	35,545,248 (91.91%)	90.84	28,562,447 (80.36%)	27,047,544 (94.70%)
B1	40,011,542	36,950,600 (92.34%)	91.08	29,530,300 (79.92%)	28,227,592 (95.59%)
B2	42,082,072	38,694,198 (91.94%)	89.80	30,691,892 (79.32%)	29,280,727 (95.40%)
B3	43,723,232	40,563,542 (92.77%)	91.24	32,648,377 (80.49%)	31,192,551 (95.54%)
C1	42,767,546	39,166,056 (91.57%)	91.52	31,415,529 (80.21%)	29,824,376 (94.94%)
C2	43,187,530	39,626,174 (91.75%)	91.14	31,744,384 (80.11%)	30,137,984 (94.94%)
C3	41,563,920	38,298,794 (92.14%)	90.59	30,513,325 (79.67%)	28,991,127 (95.01%)
D1	42,163,278	38,866,774 (92.18%)	91.29	31,243,679 (80.39%)	29,832,406 (95.48%)
D2	41,325,128	38,027,950 (92.02%)	91.07	30,559,996 (80.36%)	29,179,418 (95.48%)
D3	40,312,100	37,029,356 (91.85%)	91.05	29,625,922 (80.01%)	28,282,098 (95.46%)

Sample: petal sample’s name; Raw Reads: the total number of original reads before filtering; Clean Reads: the remaining reads after filtering, where the percentage in parentheses represents clean reads relative to raw reads; Q30 (%): the proportion of bases with a base recognition error rate of 0.1% or less; Total Mapped: the total number of reads mapped to the reference genome, where the proportion in parentheses is total mapped/clean reads; Uniquely Mapped: the reads which can be mapped to the reference genome at only one site, where the proportion in parentheses is uniquely mapped/total mapped.

**Table 3:** Summary of functional annotation of transcripts in the five public databases searched.

Annotated Databases	Gene Number	Matching Proportion (%)
Nr	28,273	85.68
Swiss-Prot	18,054	54.71
Pfam	24,301	73.64
GO	19,099	57.88
KEGG	11,507	34.87

**Table 4:** Gene expression levels of anthocyanin biosynthesis in pairwise comparisons.

Id	log_2_(Fold Change)			Description
	D vs. A	C vs. A	B vs. A	
*Rhsim04G0145800*	1.02		1.00	PAL
*Rhsim01G0211600*	1.44	1.68		4CL
*Rhsim07G0135800*			−1.08	4CL
*Rhsim13G0208200*		−5.79		F3′5′H
*Rhsim04G0208200*		−2.05		F3′5′H
*RhsimUnG0095500*	2.12	1.75	1.52	FLS
*RhsimUnG0143300*	3.35	2.01	3.29	FLS
*RhsimUnG0134400*	2.97		1.68	FLS
*Rhsim04G0219900*		3.64		FLS
*RhsimUnG0007900*	1.18		2.15	FLS
*RhsimUnG0105700*			3.54	FLS
*Rhsim12G0144700*	2.70	2.94	3.48	Leucoanthocyanidin reductase
*Rhsim06G0030600*	−4.36	−4.22	−6.99	DFR
*Rhsim06G0030500*	−1.22	−2.45	−2.55	DFR
*Rhsim06G0030400*		−1.15	−1.30	DFR
*Rhsim07G0096600*			1.33	ANS

Note: Blank present in the table indicates that there is no significant difference in gene expression.

## Data Availability

The data is contained within the article and Supplementary Materials.

## Supplementary Materials

The following are available online at https://www.mdpi.com/article/10.3390/plants12142656/s1. Figure S1: The distribution and number of DEGs in three main categories in the three pairwise comparisons; Figure S2: Enrichment of top 10 GO pathways; Figure S3: GO enrichment analysis of DEGs in the three pairwise comparison groups; Figure S4: Distribution of the genes involved in phytohormone metabolic pathways in the three pairwise comparisons. Table S1: The contents of anthocyanin components; Table S2: Primer sequences of genes used in qRT-PCR validation; Table S3: Significantly enriched GO terms in the three pairwise comparison groups; Table S4: Genes involved in the anthocyanin biosynthesis pathway showing genotypic difference expression; Table S5: List of differentially expressed TFs identified in the three pairwise comparisons; Table S6: List of phytohormone signal-transduction- and biosynthesis-related DEGs.
