# Supplementary material for: Transcriptomic Analysis Reveals the Regulatory Mechanism of Color Diversity in Rhododendron pulchrum Sweet (Ericaceae)
Source: Plants (Basel). 2023 Jul 15;12(14):2656. doi: 10.3390/plants12142656 (PMC10384940; doi:10.3390/plants12142656)
Supplement: Supplementary file 1 [file plants-12-02656-s001.zip › Figure S2. Enrichment of top 10 GO pathways.pdf]

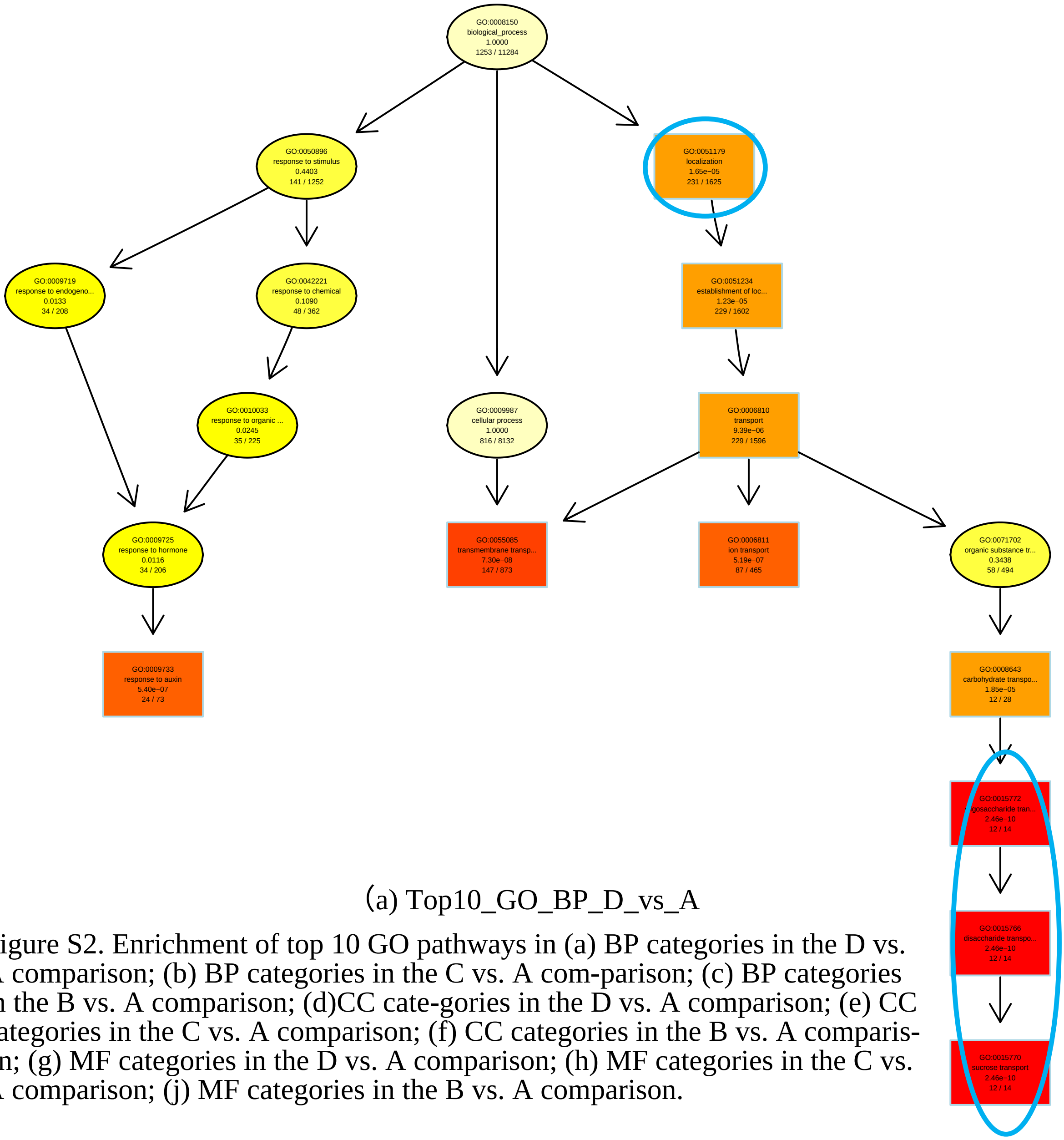


(a) Top10_GO_BP_D_vs_A

Figure S2. Enrichment of top 10 GO pathways in (a) BP categories in the D vs. A comparison; (b) BP categories in the C vs. A com-parison; (c) BP categories in the B vs. A comparison; (d)CC cate-gories in the D vs. A comparison; (e) CC categories in the C vs. A comparison; (f) CC categories in the B vs. A comparis-on; (g) MF categories in the D vs. A comparison; (h) MF categories in the C vs. A comparison; (j) MF categories in the B vs. A comparison.

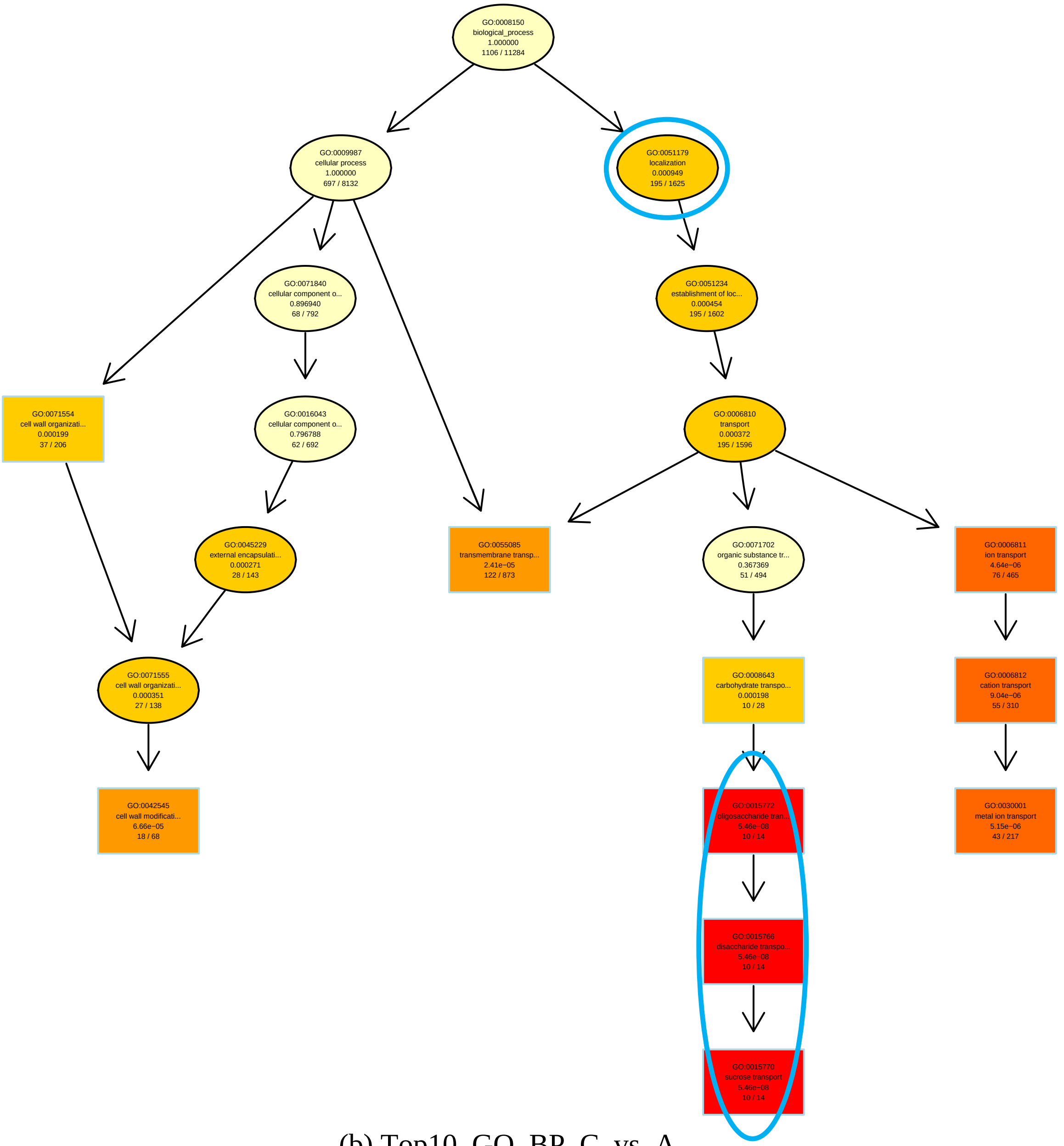


(b) Top10_GO_BP_C_vs_A

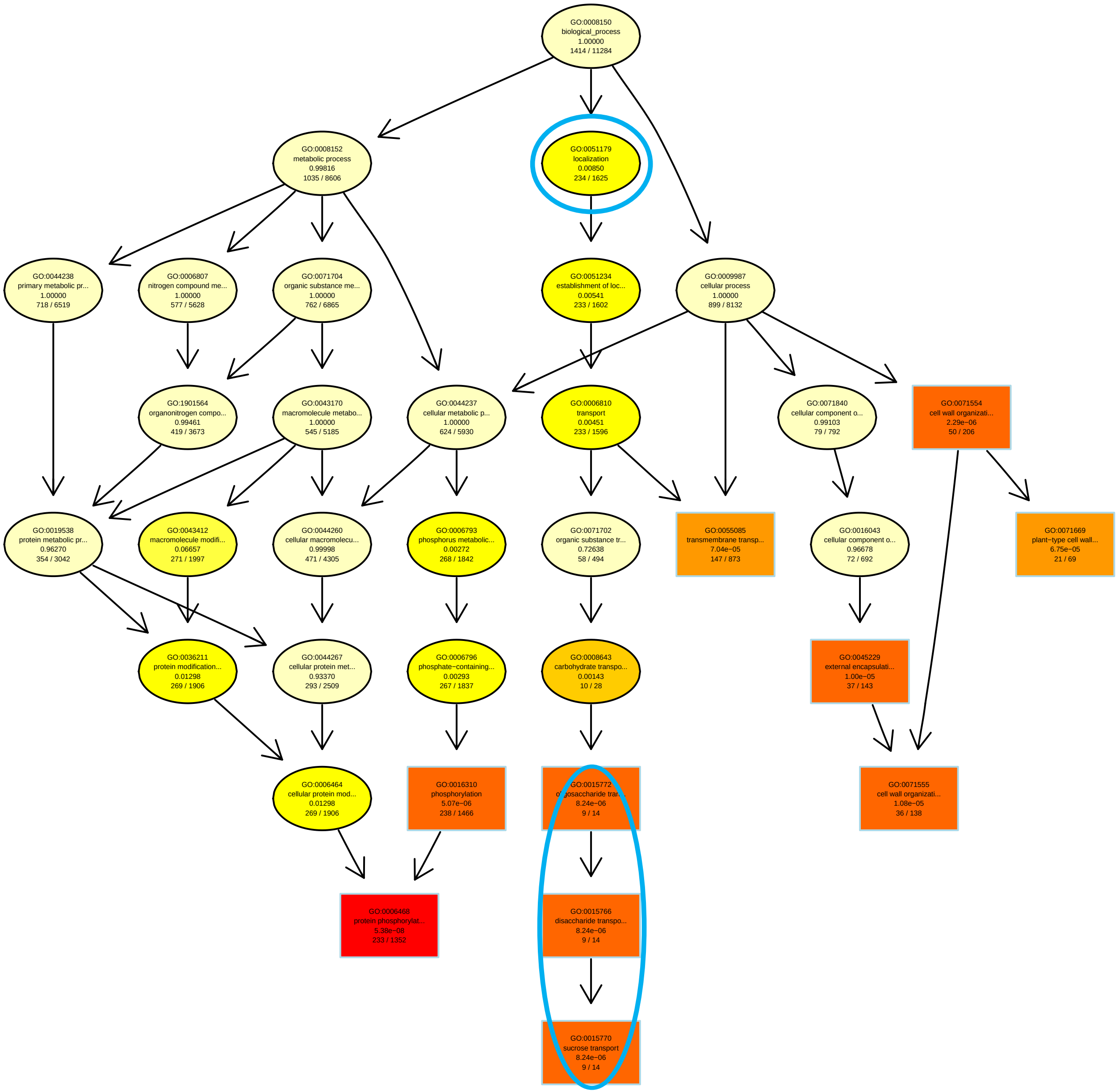


(c) Top10_GO_BP_B_vs_A

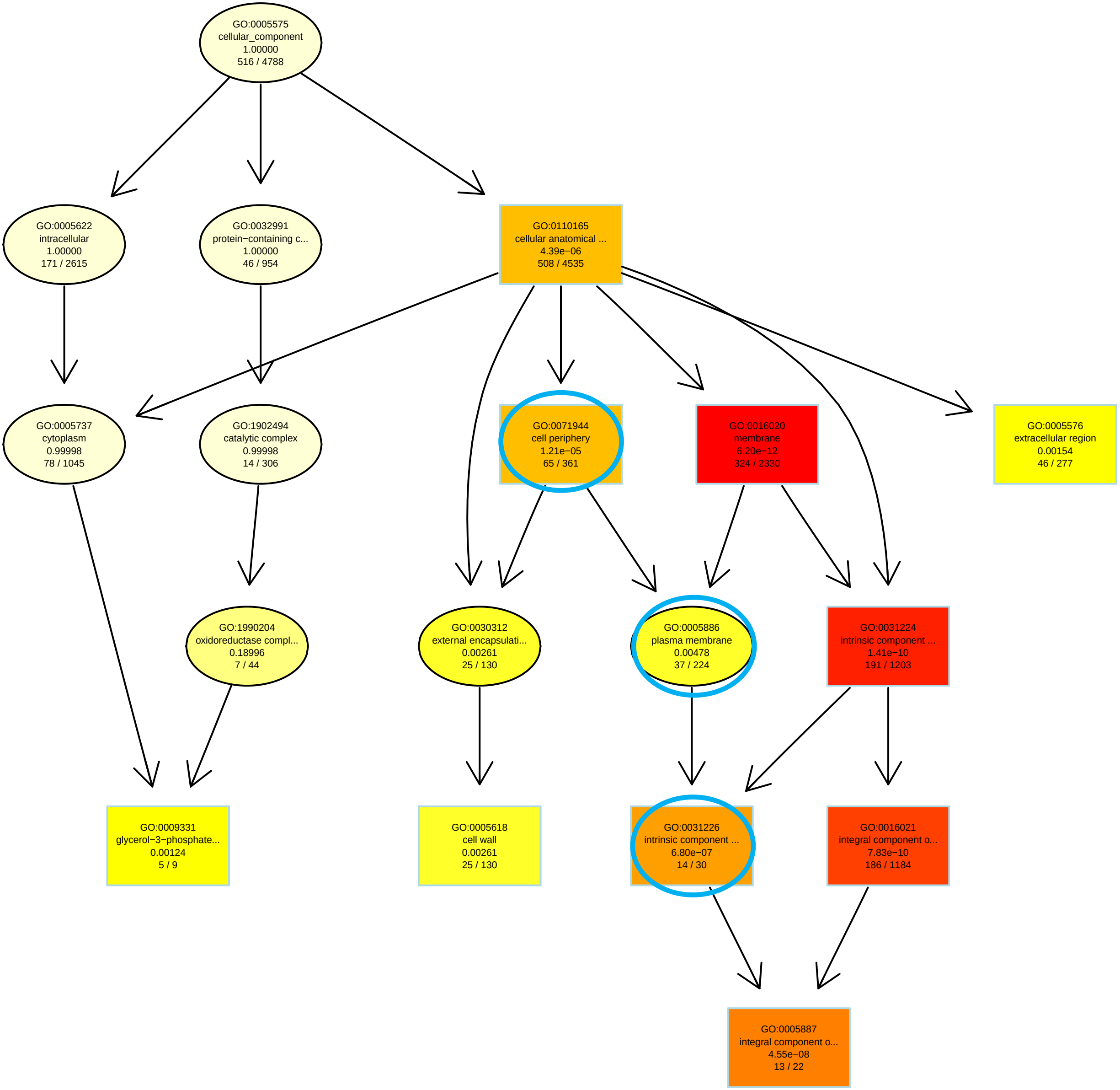


(d) Top10_GO_CC_D_vs_A

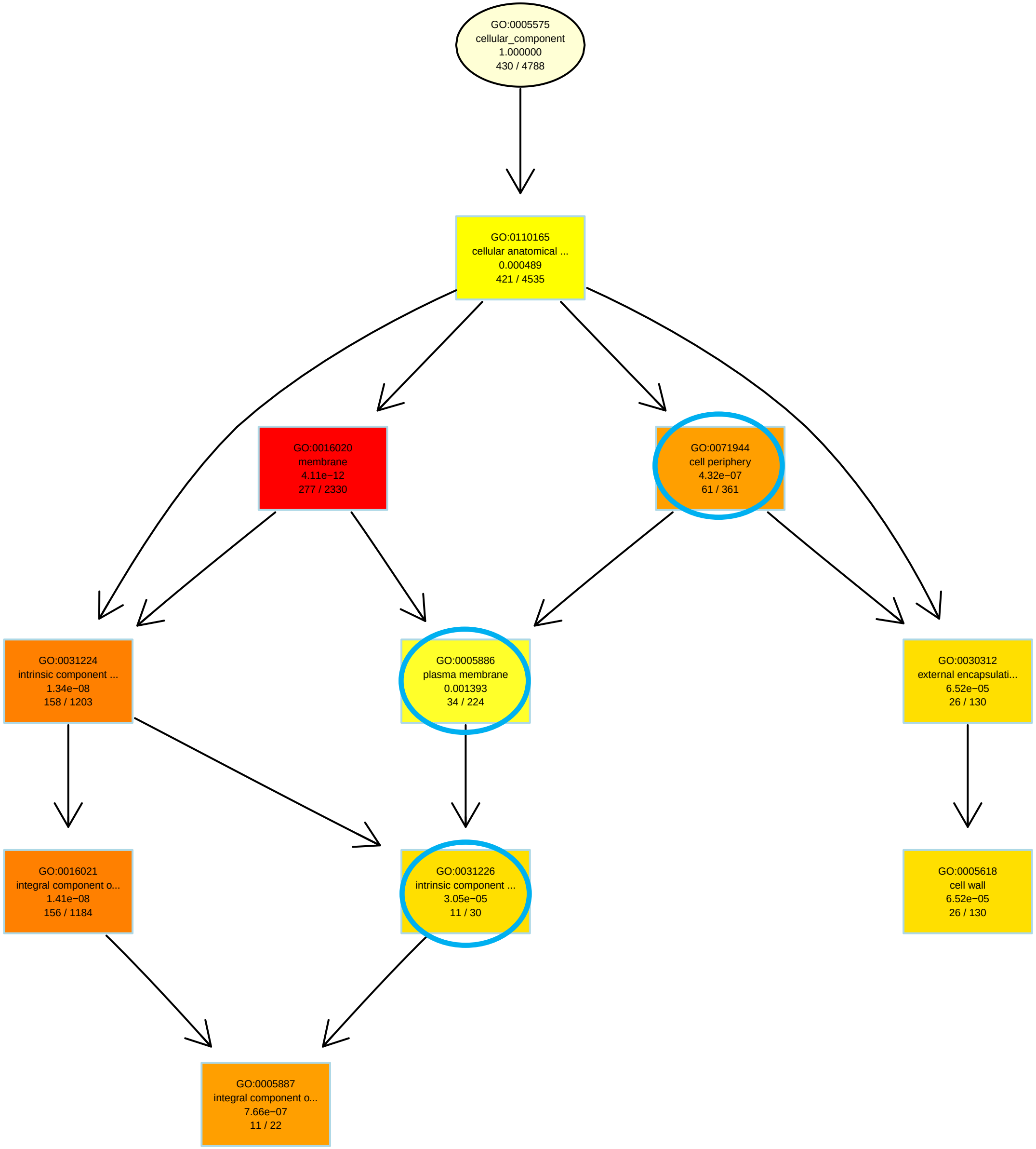


(e) Top10_GO_CC_C_vs_A

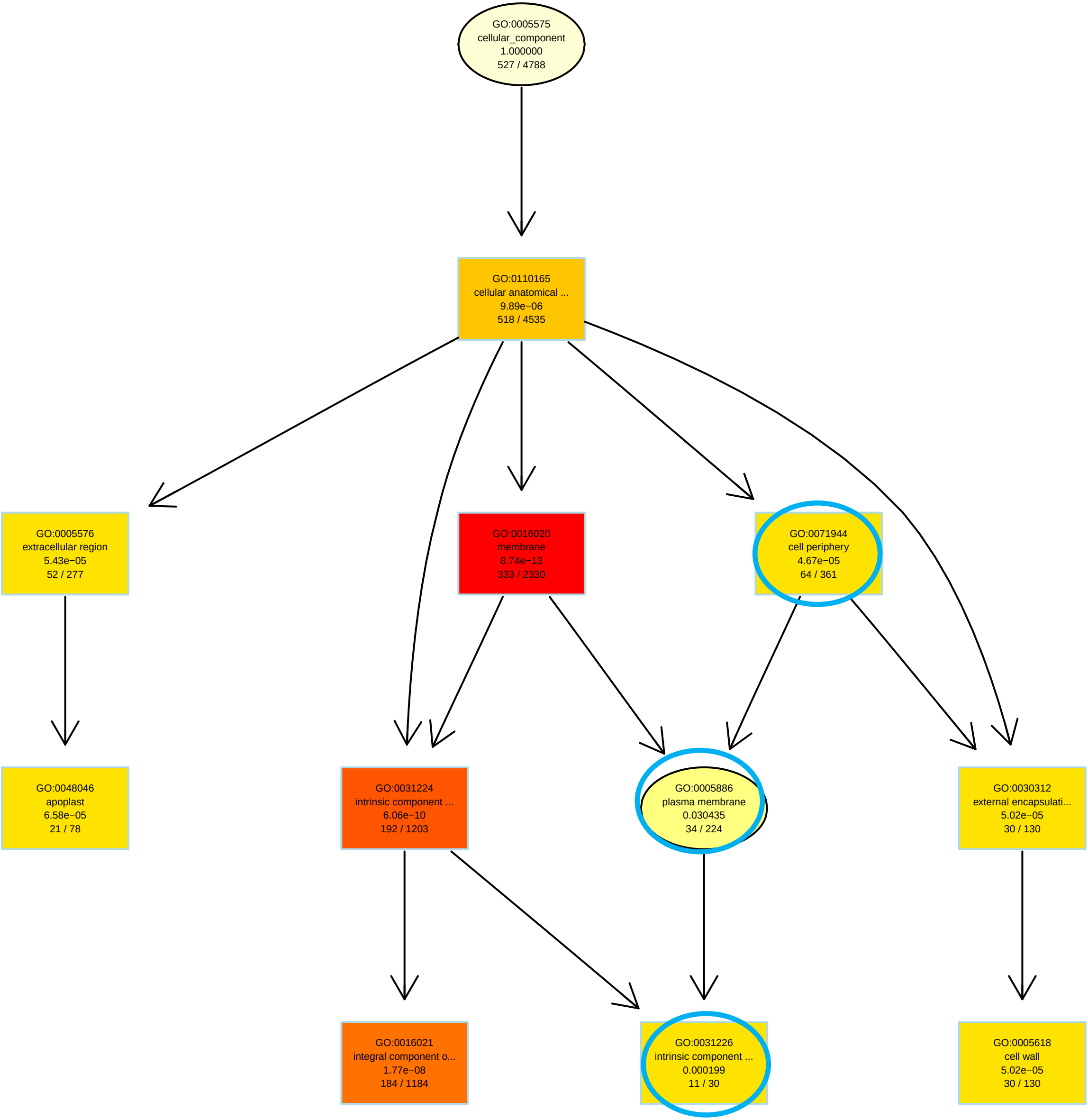


(f) Top10_GO_CC_B_vs_A

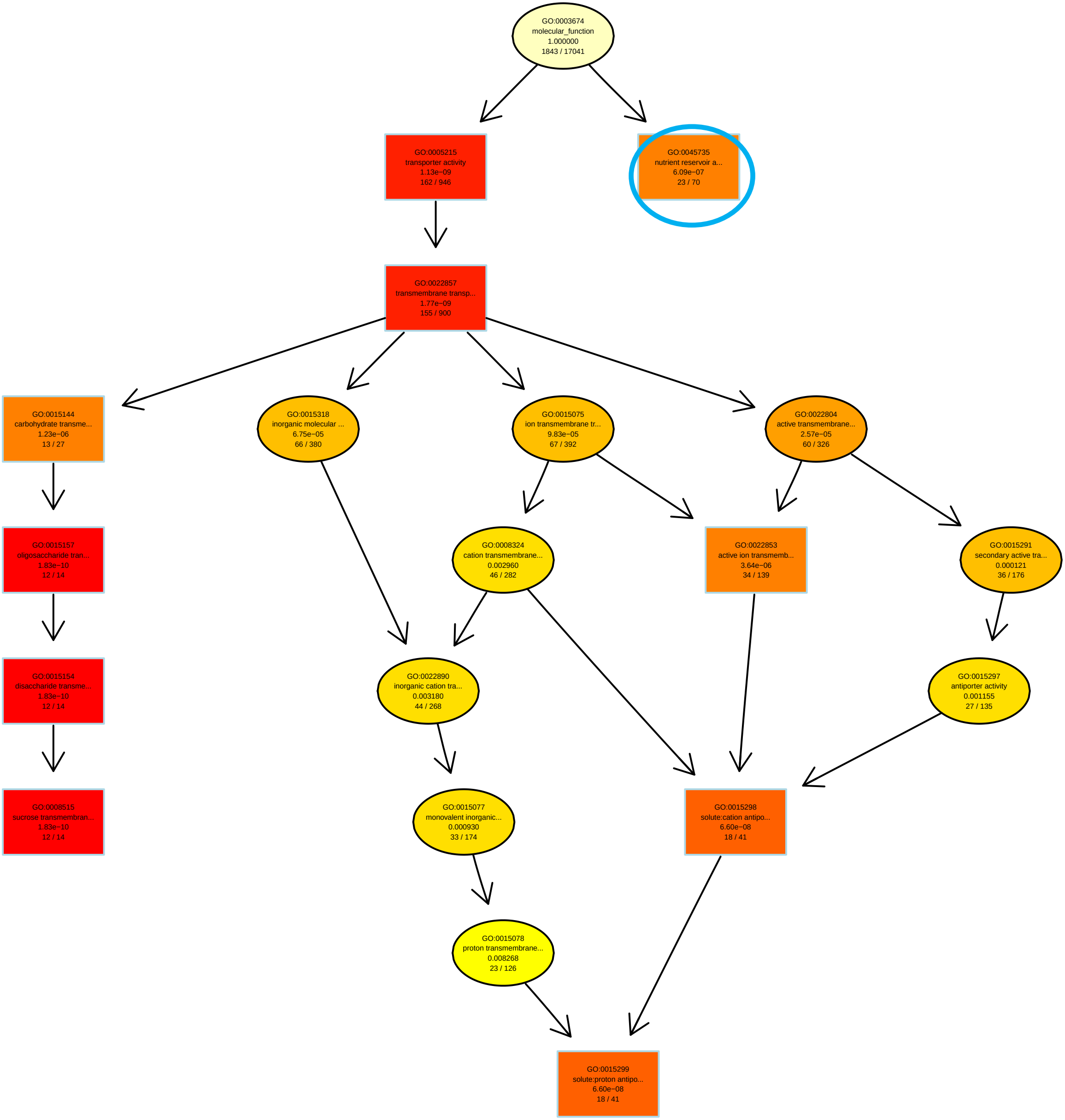


(g) Top10_GO_MF_D_vs_A

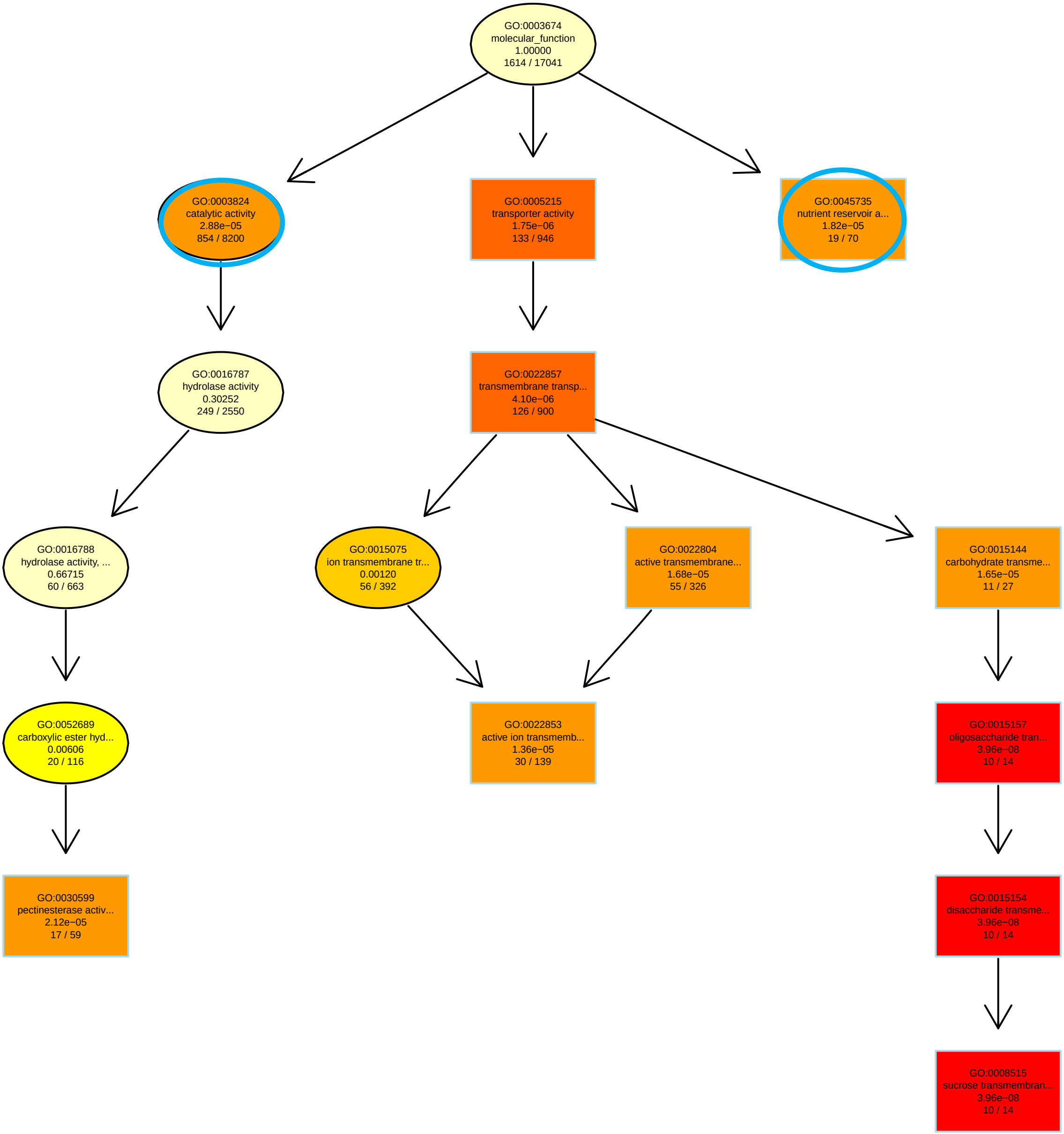


(h)Top10_GO_MF_C_vs_A

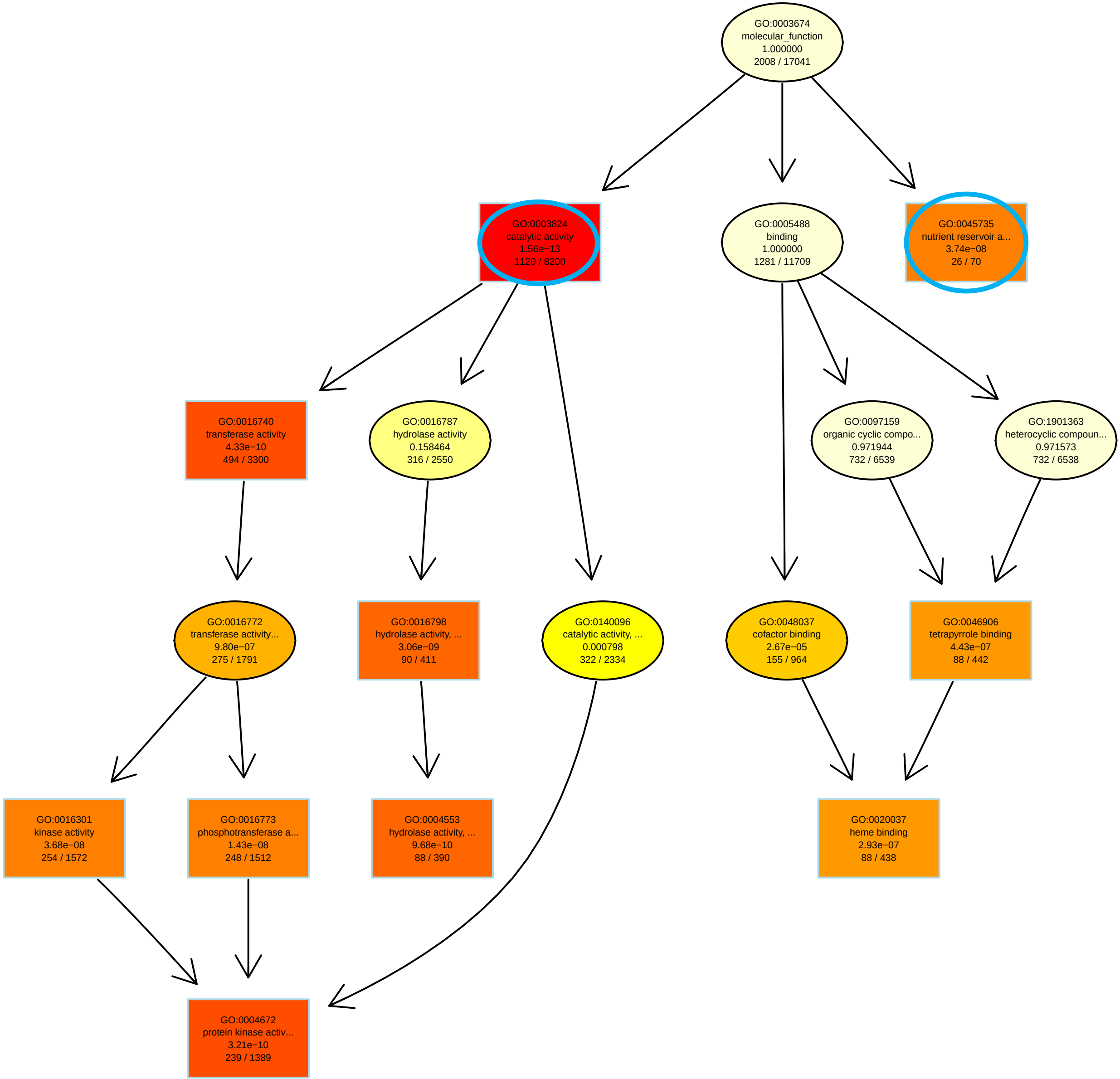


(i) Top10_GO_MF_B_vs_A
