# Supplementary material for: Transcriptomic Analysis Reveals the Regulatory Mechanism of Color Diversity in Rhododendron pulchrum Sweet (Ericaceae)
Source: Plants (Basel). 2023 Jul 15;12(14):2656. doi: 10.3390/plants12142656 (PMC10384940; doi:10.3390/plants12142656)
Supplement: Supplementary file 1 [file plants-12-02656-s001.zip › plants-2369245 Supplementary Figures S1, S3, S4.pdf]

Supplementary Materials

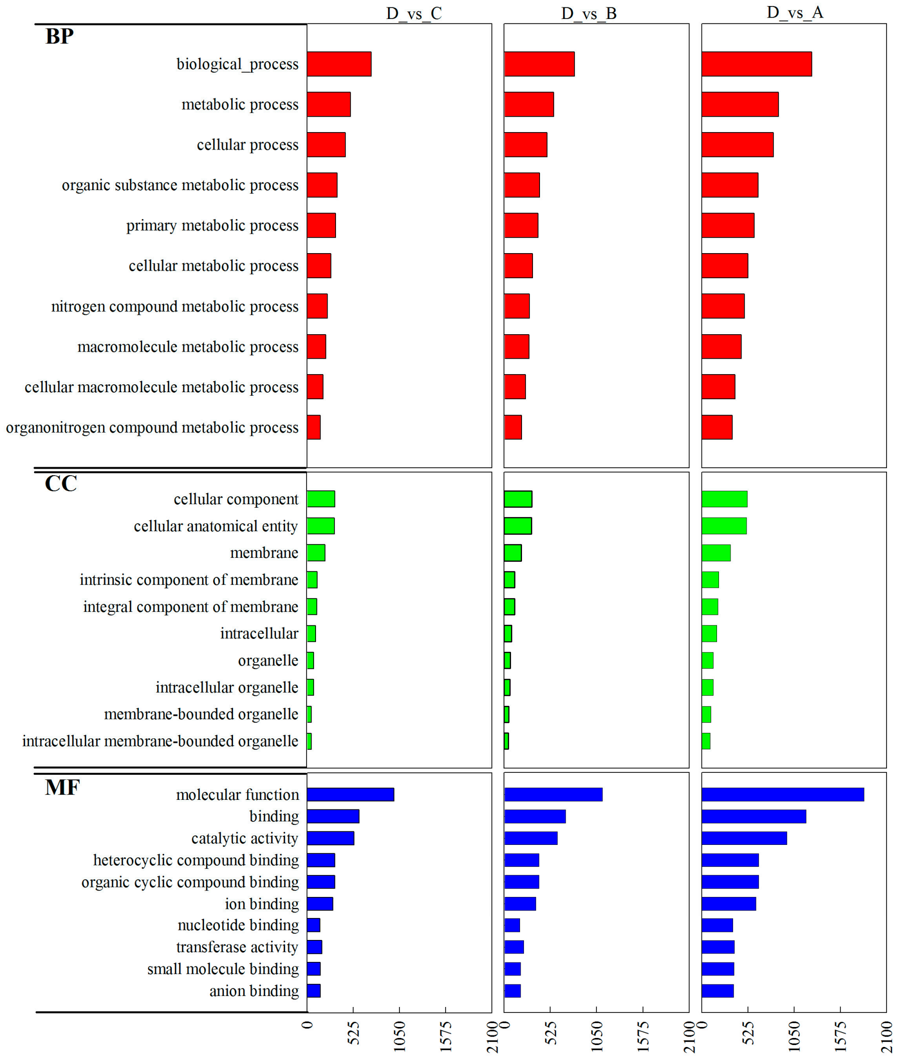

Figure S1. The distribution and number of DEGs in three main categories in the three pairwise comparisons. BP: biological process, CC: cellular component, MF: molecular function.

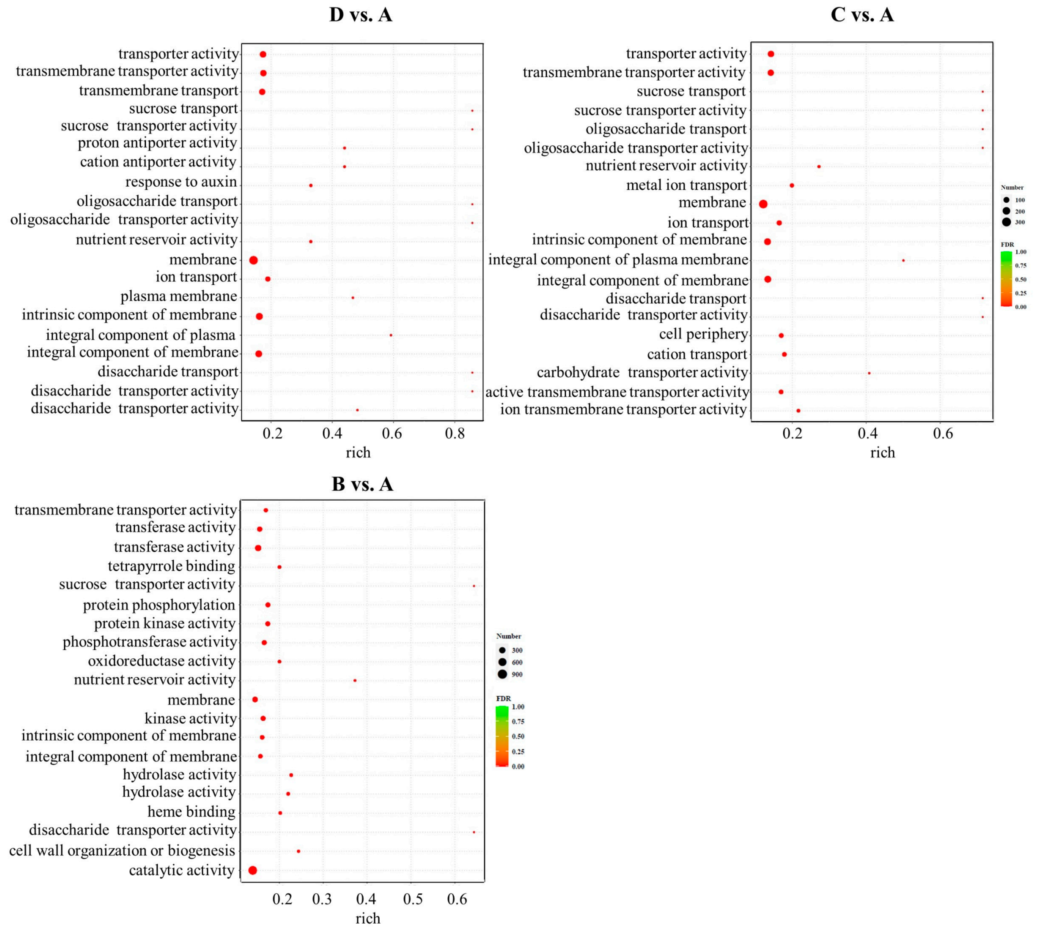

Figure S3. GO enrichment analysis of DEGs in the three pairwise comparison groups. The size of the dot indicates the number of DEGs and the color represents the *q*-value of the enrichment. The coloration scale and annotation are shown on the right side of the figure.

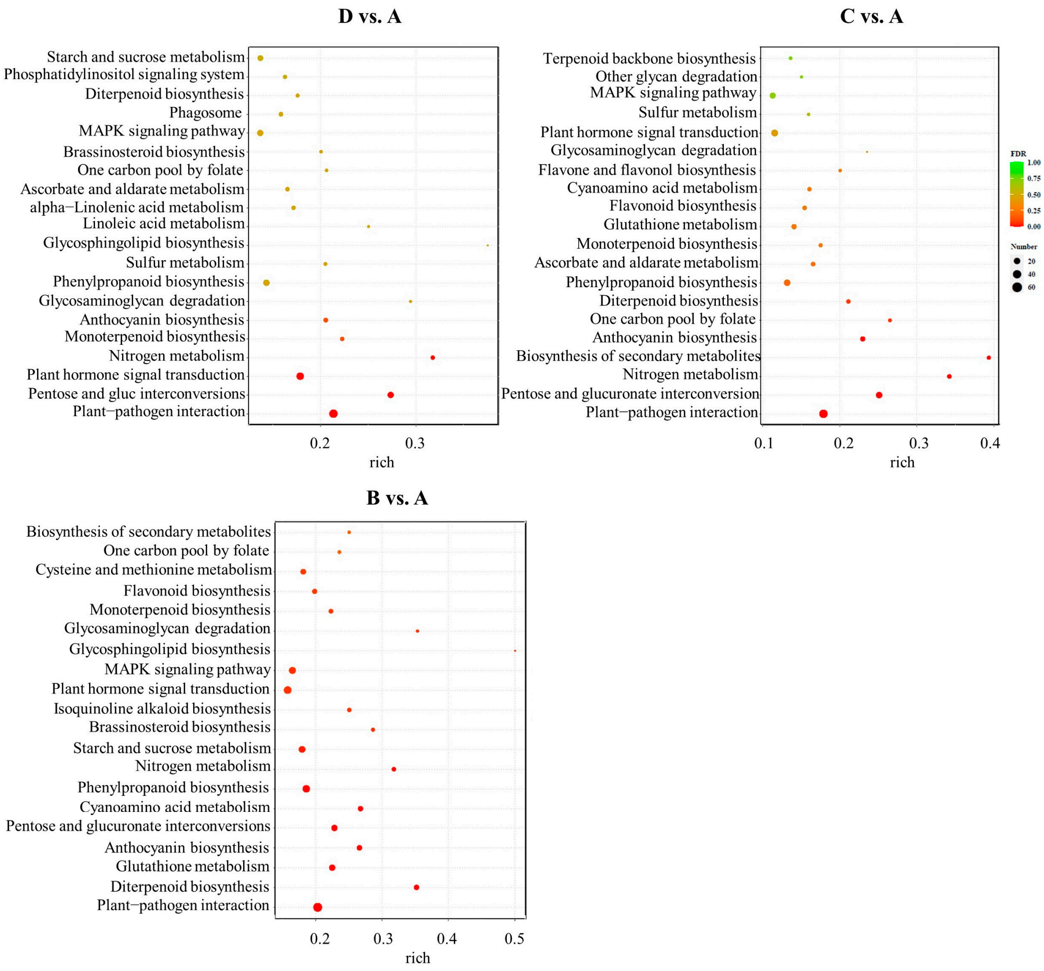

Figure S4. KEGG pathway enrichment analysis of the identified DEGs among the three comparison groups. The x-axis represents the rich factor, the y-axis indicates the name of the KEGG pathway. The size of the dot indicates the number of DEGs in each pathway while dot color represents the q-value of the enrichment.
